# High Rates of Honey Bee Colony Losses and Regional Variability in Ethiopia Based on the Standardised COLOSS 2023 Survey

**DOI:** 10.3390/insects15060376

**Published:** 2024-05-22

**Authors:** Teweldemedhn Gebretinsae Hailu, Alem Tadesse Atsbeha, Kibebew Wakjira, Alison Gray

**Affiliations:** 1 Department of Livestock Population Genomics, Institute of Animal Science, University of Hohenheim, Garbenstr 17, 70599 Stuttgart, Germany; teweldemedhng.hailu@uni-hohenheim.de; 2 Department of Animal Sciences, Aksum University, Shire P.O. Box 314, Ethiopia; lemtt2008@gmail.com; 3 Holeta Bee Research Centre, Holeta P.O. Box 22, Ethiopia; wkibebew@gmail.com; 4 Department of Mathematics and Statistics, University of Strathclyde, 26 Richmond Street, Glasgow G1 1XH, UK

**Keywords:** colony loss, varroa, beekeeping, natural disaster, honey bee

## Abstract

**Simple Summary:**

In addition to invaluable ecosystem services, beekeeping offers opportunities for job creation, income generation and food security. Beekeepers have been experiencing economic losses due to high rates of honey bee colony losses driven by various factors worldwide. We conducted this study using the COLOSS monitoring survey tools for the first time in Ethiopia to assess honey bee colony loss rates, annual colony development, beekeeping practices, and to determine the role of management practices (varroa monitoring, varroa treatment, colony splitting, feed supplementation, use of natural comb, and purchase of beeswax from external sources), as well as region, on colony losses. For this, data were collected by interviewing beekeepers from two major beekeeping regions in the country—Oromia and Tigray. Our results showed a high rate of colony losses in Ethiopia, which significantly varied between the regions. The main drivers of honey bee colony losses are related to natural disasters (particularly war), beekeeping husbandry practices, and pest management. Therefore, it is important to promote the capacity of smallholder beekeepers to implement improved beekeeping practices such as feed supplementation, queen replacement and pest management that would lead to reduced losses, increased profitability, and improved food security and livelihood.

**Abstract:**

The COLOSS research association has been assessing honey bee colony losses, associated risk factors and management, focusing on Western countries but with a progressive international expansion. Here, we report the first survey on the loss rates of colonies in 2022/2023 in Ethiopia using COLOSS monitoring survey tools. A face-to-face interview questionnaire survey was conducted on 64 beekeepers selected from Oromia and Tigray regions. This covered 1713 honey bee colonies distributed in 68 apiaries. The percentages of colonies lost were significantly different between Oromia (24.1%) and Tigray (66.4%) regions. Colony losses were attributed as unsolvable queen problems (8% in Oromia; 10% in Tigray), natural disaster (32%; 82%), and empty hives or dead colonies (60%; 8%). The loss rate was significantly affected by queen replacement (*p* < 0.0001), use of natural comb (*p* < 0.0001), feed supplementation (*p* < 0.0001), region (*p* < 0.0001), varroa treatment (*p* < 0.0001), colony splitting (*p* < 0.01), and merging (*p* < 0.01). Beekeepers in Oromia managed more colonies and implemented improved practices compared to those in Tigray. However, all beekeepers in Oromia detected at least some bees with signs of deformed wing virus, compared to 76% of beekeepers in Tigray. In conclusion, the colony loss rate was significantly different between Oromia and Tigray regions due to differences in natural disasters, management, environment and health factors.

## 1. Introduction

Following reports on high rates of colony losses and the recognition of colony collapse disorder [1,2], assessing colony losses, their driving factors and management strategies have been among major research agendas worldwide since the last decade. International survey reports from COLOSS showed overall annual loss rates of 16% to 20.9%, which greatly varied from 2% to about 36.5% between countries [3,4,5,6]. A recent study from the Bee Informed Partnership in the United States indicates up to 50.8% average loss rate of colonies [7]. The ectoparasite *Varroa destructor* (“varroa” below) is one of the major causes of colony losses [8,9,10,11]. However, some honey bee populations have been identified as tolerant against this pest and able to survive even without anti-varroa treatment [12,13,14,15]. Since the detection of varroa for the first time in northern Ethiopia’s Tigray region [16], several studies have been conducted on its distribution and prevalence in the country [17,18,19]. A national survey conducted in Ethiopia, which covered all regions except Tigray, showed a wide distribution and up to 95.8% prevalence in the country with a significant variation between regions [18]. The prevalence of varroa in Ethiopia was reported to be influenced by several factors including agroecology and management systems [19].

Other factors that cause honey bee colony losses include pesticides, natural disasters and climatic factors globally [4,20,21]. Recently, it has been discussed that climate change could significantly affect honey bees and beekeeping by causing extreme weather, flooding, wildfire, increased pest infestation and reduced forage availability [22,23]. Climate change affects vegetation distribution and the annual flowering calendar of plants, which varies between habitat elevations [24]. This can directly affect survival and performance of honey bees, as they depend on flowering plants. African honey bees are known for strong migratory behaviour, following forage seasonality [25]. In northern Ethiopia, a high rate of annual fluctuation in the numbers of managed honey bee colonies has recently been reported. Colony selling and losses (including absconding, death and seasonal migration) were identified as the reasons for colony outflow in the areas, while purchase, trapping, splitting and swarming are the mechanisms of inflow [26]. Honey bee colonies are also valuable trading commodities in northern Ethiopia where beekeepers exchange colonies in central markets [27]. A high rate of mobility of honey bee colonies under the influence of anthropogenic activities such as colony marketing, as well as natural circumstances (such as migratory behaviour, habitat fragmentation or rehabilitation) could exacerbate the distribution of varroa and other pests and pathogens [28]. Honey bee colony transportation across agroecological zones is a common practice in northern Ethiopia’s Tigray region [27,29].

Tigray region is known as one of the major beekeeping centres in Ethiopia, where there has been a pronounced transformation of beekeeping. Data from the Central Statistical Agency of Ethiopia [30] showed the management of more than 340,000 honey bee colonies in the region. Over the last two decades, the percentage of frame hives has grown from 1% to 23% and the number of managed colonies has increased by 90% [31]. However, in recent years, Ethiopia has been facing difficulties due to war, COVID-19, climate change and outbreaks of *Desert locust* [32,33,34]. In particular, the two-year war from November 2020 to November 2022 in Tigray region was reported to have wiped out decades of progress in all sectors [35,36,37,38]. Considering beekeeping specifically, 70% of the managed honey bee colonies were lost due to the war [39]. This loss may threaten both the livelihoods of the beekeepers and the overall ecosystem.

A survey conducted during the pre-war period in northern Ethiopia showed that there was no difference in colony loss rates based on the beekeeping system, beekeeping experience, number of colonies per household and local areas within Tigray [26]. This differs from international surveys, which have reported that loss rates are significantly affected by the scale of beekeeping operations, geographic location and climate, although Ethiopia was not included in these studies [3,4,5,6,20,21]. Therefore, this study was conducted to assess the loss rates in different regions using the COLOSS survey protocol for the first time. The hypothesis is that honey bee colony loss rate significantly varies between regions depending on the vulnerability to natural disasters, infestation and prevalence of varroa, and beekeeping practices, as reported by the international surveys. The alternative hypothesis would be that there is no difference in honey bee colony loss rates between different regions in Ethiopia, as previously found for different local areas within Tigray. The specific objectives of this survey were to assess honey bee colony loss rates in the Oromia and Tigray regions of Ethiopia and to evaluate the degree of association between honey bee colony losses with colony management practices (varroa monitoring, varroa treatment, colony splitting, feed supplementation, operation size, use of natural comb, and purchase of beeswax from external sources). The results are discussed with reference to regional and global figures for colony losses, based on the literature.

## 2. Materials and Methods

### 2.1. Study Regions and Description

**Figure 1:**
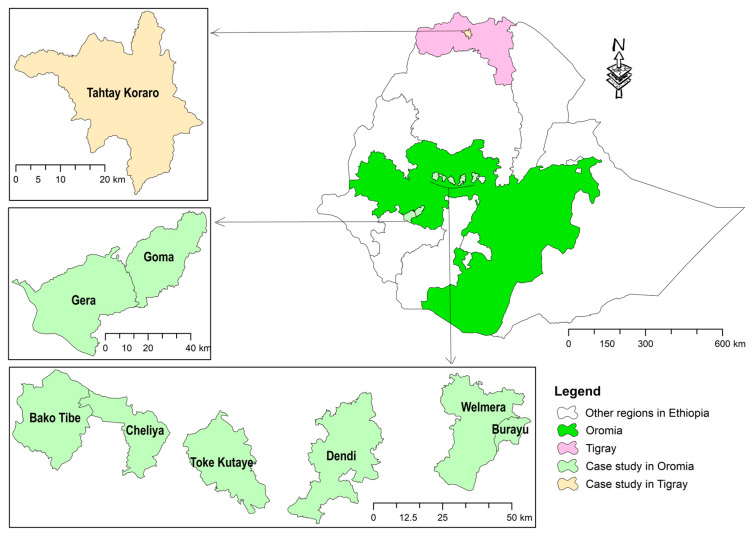
Location map of surveyed districts in Oromia and Tigray Regional States of Ethiopia in 2023.

This survey was conducted in Ethiopia, namely Oromia and Tigray Regional States (Figure 1). Tigray is predominantly a semi-arid region located in northern Ethiopia, which is known as a main beekeeping area in the country, and for its famous white honey [40], unique colony marketing system [29] and a rapid beekeeping transformation in the last two decades. In this region, *Varroa destructor* was detected in 2010 for the first time in Ethiopia [16]. From November 2020 to November 2022, the region was heavily devastated by war that has caused significant damage also to the beekeeping sector [39]. On the other hand, Oromia is the largest region in Ethiopia, connects with almost all regions and is largely located in the central, southern and western parts of the country. Likewise, Oromia hosts the largest number of honey bee colonies in the country [30]. Our study areas in Oromia are mainly located in the west and southwest, which are largely humid.

Therefore, the survey was conducted in these two important beekeeping regions characterised by contrasting features of beekeeping, climate, vegetation, etc.

### 2.2. Survey and Data Collection

For this study, 64 beekeepers who managed a total of 1713 honey bee colonies during the survey year 2022/2023 were purposefully selected based on accessibility. The colonies were distributed over 68 apiaries and 14 villages (locally called *Kebelles*) of the two regions (Table 1). Data collection was conducted by interviewing the beekeepers face-to-face using the standardised questionnaire of the COLOSS Colony Losses Monitoring Core Project. Therefore, the results are comparable to those from other countries using the same questionnaire, in particular the loss rates. Interviews were conducted by researchers who orally translated the questionnaires from English to local languages directly when asking the beekeepers. As is established practice in Ethiopia, the aim of the survey was explained verbally to the selected households/beekeepers, who then responded to the questions voluntarily. This data collection was performed during June 2023.

### 2.3. Statistical Analysis

Variables analysed and included in this paper are comprised of: (1) number of honey bee colonies managed (as a measure of beekeeping scale of operation), honey bee colony losses and loss rates; (2) beekeeping practices such as colony splitting, feed supplementation, queen replacement, and colony merging; (3) honey bee health, monitoring and pest management; (4) annual honey bee colony cycle and management calendar. These data were analysed and comparisons were made between the Oromia and Tigray regions. As the number of colonies before winter and the number of colonies in spring 2022 and spring 2023 have very skewed distributions, medians are reported as well as means, and Mann–Whitney tests are used to compare the numbers in the two regions. Fisher’s exact test was used to compare the proportions of beekeepers giving each response to the management questions between regions. The data for several variables included responses in the categories labelled as “Don’t know” or “Not applicable”. These categories of responses were not removed for the purposes of analysis, but retained. For two variables (monitoring varroa and observing bees with deformed wings), the numbers of responses in this category were very low (Table 2) compared to other responses and were similar for the two regions, so would not affect the conclusions. For two other variables, concerning treating varroa and presence of *Vespa velutina*, there were more responses of “Not applicable” and “Don’t know”, respectively, for one region and very few for the other; this is discussed further in Results.

Colony loss rates are reported as the overall proportion of colonies lost, with a 95% confidence interval, and also as the average rate of loss per beekeeper. Colony loss rates per beekeeper can be assumed to be normally distributed for Oromia (Anderson–Darling test, *p* > 0.05), but not for Tigray (*p* < 0.05). Therefore, colony loss rates per beekeeper were compared between the regions using the Mann–Whitney test.

Furthermore, correlation analyses were performed to assess the degree of associations between the different components of colony losses (queen problems, natural disaster, dead colonies, empty hives) and the numbers of colonies managed at different seasons of the survey year.

Intercept-only quasibinomial generalised linear models (GLMs) were used to obtain confidence intervals (CI) for the proportional loss rates, and univariate and multivariate quasibinomial GLMs were used to assess the significance of risk factors for colony loss for both regions combined and also separately. In particular, an F-test from a univariate quasibinomial GLM, or a Chi-squared test of proportions, allowed comparison of the proportional loss rates between regions. The R software version 3.6.3 [41] and JMP Pro 17 [42] were used for the data analyses.

## 3. Results

### 3.1. Colony Management in Oromia and Tigray Regions

**Figure 2:**
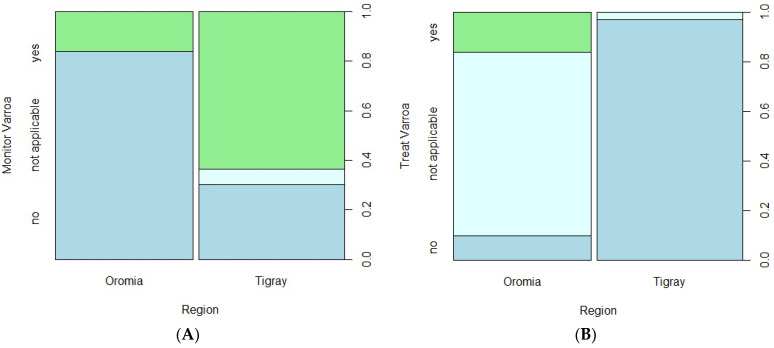
Varroa monitoring (**A**, left) and treatment (**B**, right) in Oromia and Tigray regions of Ethiopia during 2022/2023 Colony Losses Monitoring Core Project survey year of COLOSS. The columns for each region in plots A and B are split between the response categories “no” (shown in mid-blue), “not applicable” (light blue) and “yes” (green) of the factor shown on the vertical axis; the relative split of the columns shows the proportions of the respondents in each category and allows comparison of the responses between the regions.

Table 2 shows a summary of descriptive results for the two studied regions and hypothesis tests comparing the regions. Beekeepers included in this study managed an average number of colonies of 27.00 and 9.03 in the Oromia and Tigray regions, respectively, in spring 2022. These increased to about 42.10 and 12.36 colonies on average before winter 2023 and again declined to 30.58 and 5.24 in spring 2023, respectively. Correlation analyses indicated that the number of colonies in spring 2022 and spring 2023 were strongly associated (Spearman correlation: *r* = 0.92, *p* < 0.0001). Mann–Whitney tests showed that there were significantly higher numbers of honey bee colonies in the Oromia region than in Tigray region during both 2022 and 2023 years (*p* < 0.001). A significantly higher proportion of beekeepers in Tigray monitored the status of varroa in their colonies (Figure 2). On the other hand, none of the beekeepers in Tigray applied any kind of colony treatment against varroa, whereas some beekeepers (16.1%) in Oromia applied the biotechnical method, trapping varroa mites mainly in drone brood combs and discarding them to reduce infestation without chemical treatment. While the percentage treating in Oromia is low, most of the beekeepers there (74.2%) responded that treating was not applicable.

Moreover, a significantly higher number of beekeepers in Oromia use natural combs (*p* < 0.0001) and are less dependent on externally purchased beeswax (*p* < 0.01) compared to the situation in Tigray. Furthermore, there were significantly more frequent applications of improved husbandry practices such as merging of weak colonies (*p* < 0.001), queen replacement (*p* < 0.0001), feed supplementation (*p* < 0.0001) and colony splitting (*p* < 0.0001) in Oromia. However, all beekeepers in Oromia reported the detection of some bees with deformed wings, compared to 45.5% reporting some bees with deformed wings and 30.3% reporting many bees with deformed wings in Tigray. These percentages differ significantly between the regions (*p* < 0.0001). In contrast, most beekeepers in Tigray (87.9%) observed the presence of *Vespa velutina* in their apiaries, which differs from the situation in Oromia (25.8%), although in Oromia 32.3% responded “Don’t know” to whether or not *V*. *velutina* were present (Table 2). It should be noted that these data are based on beekeepers’ description and require follow-up, particularly as *V. velutina* was not previously reported from Africa.

Furthermore, correlation analysis showed that the different components of colony losses (number of colonies lost due to queen problems, natural disaster, dead colonies or empty hives) are positively related to each of the numbers of colonies managed at different seasons of the survey year (Table 3).

### 3.2. Colony Loss Rates, Components of Loss and Risk Factors

**Figure 3:**
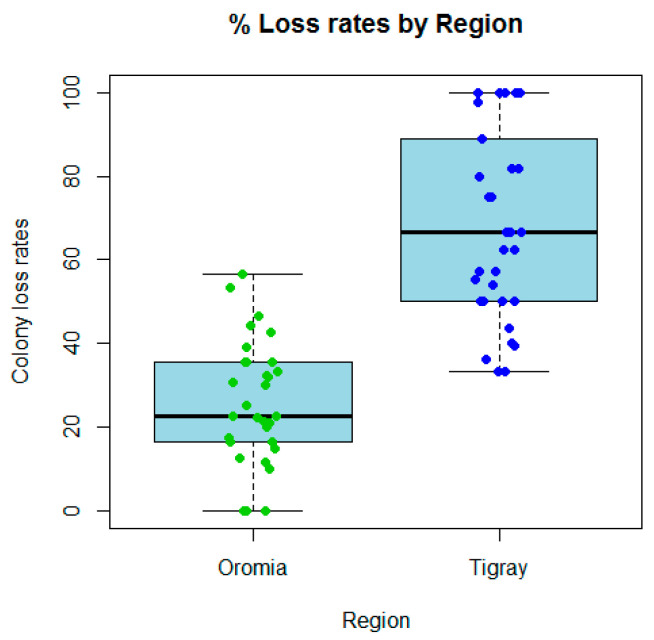
Colony loss rates (%) per beekeeper in Oromia and Tigray regions of Ethiopia, showing a markedly higher level of loss of colonies in Tigray compared to Oromia (Mann–Whitney test, *p* < 0.0001) based on the 2023 survey following the 2020–2022 war in northern Ethiopia. The individual loss rates are shown superimposed as points on the boxplots.

The average annual colony loss rate in 2022/2023 in Tigray (68.3%) was far higher than that of Oromia (25.9%), and the levels of loss per beekeeper are significantly different between these two regions in northern and central Ethiopia (Mann–Whitney test, *p* < 0.0001). The loss rate varied from 0% to a maximum value of 56.7% in Oromia, whereas in Tigray it ranged from 0% to 100% (Figure 3). Among the colonies recorded as lost in Oromia were losses due to unsolvable queen problems (8% of lost colonies), natural disaster (32%), and those found as empty hives or dead colonies (60%). The same factors but with contrasting rates of loss were reported from Tigray as queen problems (10% of lost colonies), natural disaster (82%), and empty hives or dead colonies (8%). These differences could relate to the different factors influencing colony losses in the two regions of Ethiopia as presented in the following paragraphs. Furthermore, colony absconding during the survey year was significantly higher (Fisher test, *p* < 0.01) in Tigray (97.0% reported at least one absconding event in their apiary) compared to Oromia (67.7%).

The overall proportional loss rate for all beekeepers was 34.2% across both regions, which is calculated as the total number of colonies lost by all the beekeepers divided by the total number of colonies managed by all the beekeepers and expressed as a percentage (95% confidence interval (28.5%, 40.3%)). The overall proportions lost per region were 24.1% (95% CI 19.8%, 28.9%) for Oromia and 66.4% (95% CI 58.3%, 73.7%) for Tigray. These proportional loss rates were significantly different between the two regions, *p* < 0.0001 (Chi-squared test of proportions or F-test in a univariate quasibinomial model for the risk of colony loss).

To test the effect of various factors on the risk of colony loss, quasibinomial models were fitted. In the models for both regions combined, region, varroa treatment, presence of bees with deformed wings, use of foundationless combs, queen replacement, colony splitting, merging colonies, feed supplementation, and amount of sugar supplementation were individually highly significant predictors of the risk of colony loss. In a multivariable model, region and varroa treatment were still significant, but other variables were no longer significantly associated with colony loss. Therefore, the most important predictors of colony loss were region and varroa treatment (*p* < 0.0001). In addition, models were fitted for the regions separately where there was more than one category of a factor observed in the data. For Oromia, presence of *V. velutina* and sugar supplementation were the only significant variables for predicting colony loss. Of these, only presence of *V. velutina* significantly affected the loss rate when a multivariable model was considered, and is, therefore, the most notable predictor of colony loss in Oromia (*p* < 0.01). Table S1 shows details of the best predicting models. For Tigray, none of the individual factors were significant in the fitted models, but use of foundationless combs and merging colonies were closest to being significant predictors of colony loss. These results as a whole reflect differences between the regions. Consequently, colony loss rates were significantly different between the two regions (Figure 3) due to differences in natural disasters, environmental factors and beekeeping practices. These factors include varroa monitoring, varroa treatment, use of natural combs, dependence on externally supplied beeswax, colony merging, queen replacement, feed supplementation, colony splitting and *Vespa velutina* (Table 2). In particular, the presence of *V. velutina* was the most influential factor on colony losses in Oromia. Additionally, impact of the Tigray war is a major difference between the regions.

### 3.3. Annual Colony Development and Management Calendar

**Figure 4:**
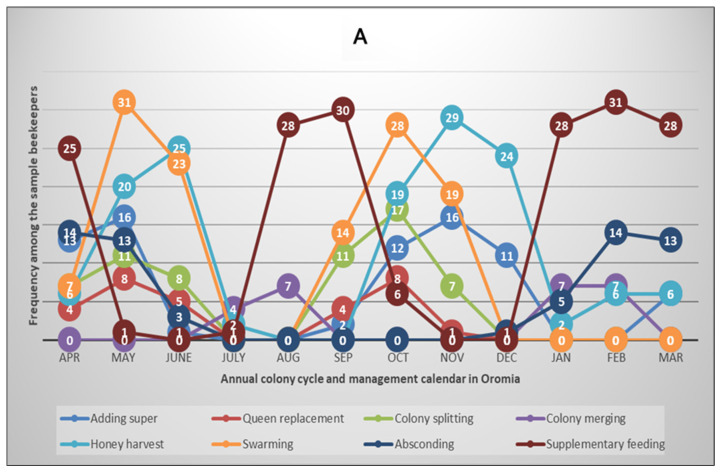

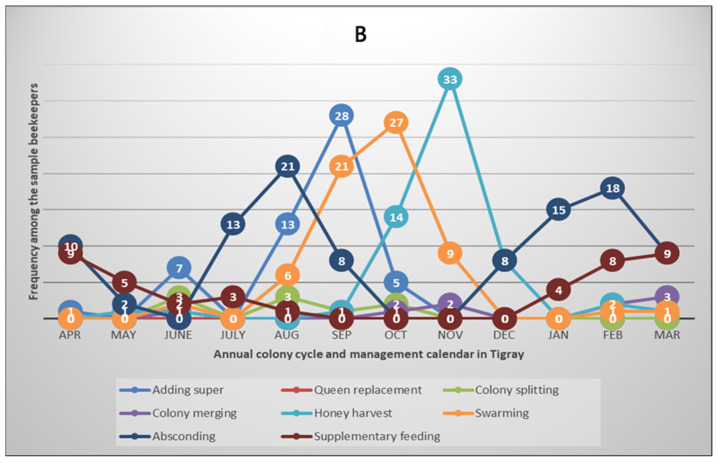
Annual honey bee colony cycle and beekeeping calendar in Oromia (**A**) and Tigray (**B**) regions of Ethiopia, northeast Africa. The plots show bimodal and monomodal patterns of colony development and the beekeeping calendar in Oromia and Tigray regions, respectively, which reflect two beekeeping seasons in Oromia and one season in Tigray annually. The numbers shown in the circles on the graphs indicate the frequency of beekeepers reporting that the specified events/activities occurred/were performed in the regions during each month of the survey year 2022/2023 of the Colony Losses Monitoring Core Project.

To obtain more insights into the annual honey bee colony development and beekeeping activity calendar, we collected data on the major events and practices that occurred and were performed by the beekeepers during the survey year. The variables include adding supers to hives, merging weak colonies, colony absconding, queen replacement, honey harvesting, feed supplementation, colony splitting and swarming. The results are summarised in Figure 4A,B for Oromia and Tigray regions, respectively.

In the Tahtay Koraro district of the Tigray region, where the survey was conducted, colonies start to develop in July and peak in September. This is followed by peak reproductive swarming and honey harvesting in October and November, respectively. After honey harvesting, about half of the beekeepers (51.5%) provide supplementary feed to their colonies in the dry months from January to May, whereas most of the beekeepers (97.0%) in this region faced at least one colony absconding event (Figure 4B, Table S2). The pattern of colony development and the beekeeping calendar in the surveyed humid areas of Oromia region was different from the situation in the semi-arid or savanna areas of Tigray region [43]. The survey results showed that there is a two-cycle (double-peak) pattern of colony development, swarming, honey harvesting, and feeding in Oromia region (Figure 4A, Table S2). Another differentiating aspect is that all sampled beekeepers practised feed supplementation during the dearth periods in Oromia region, whereas 67.7% of them faced at least one absconding event in the year compared to the 97.0% reported above in Tigray region.

## 4. Discussion

Here, we discuss the results of our survey which was conducted in Oromia and Tigray regions of Ethiopia for the first time based on the COLOSS standardised questionnaire 2023, aiming to assess honey bee colony loss rates and driving factors with reference to regional and global reports. The discussion provides insights into the factors influencing colony losses, colony management, pests, and the annual beekeeping calendar, which are shaped by anthropogenic, climatic and environmental factors.

We found higher numbers of colonies per beekeeper (apiary) in spring 2023 in Oromia (mean = 30.58) and Tigray (mean = 5.24) compared to previously reported average estimates of 6 colonies per beekeeper for Ethiopia [44] and 2 colonies per beekeeper for Tigray [26]. It should be noted that the sampling in this survey was not random, but purposefully included well-known beekeepers and accessible apiaries such as those owned by the Oromia Apicultural Research Institute, due to limitations of logistics, time and funding. Some of the apiaries were found to implement improved beekeeping husbandry practices that are not common among the Ethiopian smallholder beekeepers. These include varroa monitoring (7.8% in Oromia; 32.8% in Tigray), varroa treatment (7.8%; 0.0%), queen replacement (29.7%; 0.0%), feed supplementation (48.4%; 26.6%), merging of weak colonies (32.8%; 9.4%) and colony splitting (40.6%; 7.8%); see Table 2 and Figure 2.

Apiaries managed in Oromia that are included in this survey applied more varroa control (biotechnical method), feed supplementation, merging and splitting (*p* < 0.001) compared to those studied from Tigray. Moreover, a significantly higher number of the surveyed beekeepers in Oromia used natural combs (*p* < 0.0001) and depended less on beeswax purchased from external suppliers (*p* < 0.01) compared to the situation in Tigray (Table 2). On the other hand, most frame-hive beekeepers in Tigray depend on externally purchased beeswax supplied from other parts of Ethiopia where traditional beekeeping is still dominant and extraction of beeswax is practised. Exchanging honey bee products and colony transportation could negatively affect honey bee population structures and health [28,45,46]. Pests, pathogens and other stressors cause colony losses, as observed by survey studies in different parts of the world [3,4,5,47,48,49]. On the other hand, application of improved beekeeping husbandry such as merging and supplementing weak colonies, replacing undesirable queens, monitoring and controlling of varroa enhances the performance, health and survival of colonies [50,51,52,53], and could reduce colony losses. Altogether, colony losses can be influenced by health, nutrition, and climatic stresses as well as anthropogenic activities.

In this survey, the average loss rate of colonies in Tigray was markedly higher (68.3%) than in Oromia (25.9%). The proportional loss rate was also significantly higher in Tigray (66.4%) than in Oromia (24.1%). A previous study [26] showed that the colony loss rate in Tigray was lower (at 15.7% overall, and ranging from 9% to 19.5% between local areas) than the results in this survey and was similar when compared to international (overall proportional) loss rate figures of COLOSS reported as 16.4% to 20.9% [3,4,5]. This indicates that most of the colony losses in Tigray reported in the present study can be attributed to the disaster of the two-year war in the region, which agrees with recent reports that the Tigray war caused 70% honey bee colony losses [39] and reversed decades of ecosystem restoration [37,54], agricultural development [55,56] and livelihood improvement [34] efforts. These effects could lead to habitat degradation, shortage of bee forage and increased rates of colony absconding and death. Overall, human activities such as war, fire, vandalism and honey hunting can cause detrimental impact on the survival of honey bees and the livelihood of beekeepers.

Among the sample beekeepers, 97.0% of them in Tigray responded that they had experienced at least one colony absconding event in the surveyed year compared to 67.7% in Oromia (Figure 4, Table S2). Honey bee colony absconding can be defined as seasonal migration of the colony seeking more favourable nest and/or habitat conditions. In this behavioural adaptation, the colony abandons its old nest when there is shortage of forage, extreme weather, pest infestation and/or other disturbances [57,58,59]. Instead, the colony searches and re-establishes itself in a suitable environment. Therefore, absconding is one form of colony loss to beekeepers, although the colony may not be lost from the overall ecosystem. This is because the colony strives in another location and continues to provide the necessary ecosystem services no matter whether it may return to its original nest when the situation is better or may be trapped by another beekeeper’s bait hive. High rates of migratory and swarming behaviours in honey bees have been reported as the main coping mechanisms against pests such as varroa [60]. African honey bees are known for a high degree of migratory behaviour when the availability of floral resources is reduced [57], which could also be influenced by the local weather conditions and type of beehives [59] as well as management practices such as feed supplementation and merging of weak colonies. Therefore, colony loss rates vary with management intensity implemented by different scales of beekeeping operations, local weather, geographic location and season [3,4,5,6,20,21]. For example, in the COLOSS survey 2019/20, loss rates ranged from 7.4% in Norway to 36% in Spain, while large-scale beekeeping operations are less prone to the risk of colony losses compared to small-scale beekeepers managing fewer than 50 colonies [6]. However, this was not observed in a survey conducted in Mexico [49], which indicates the role of local/regional conditions.

Looking for annual variations in beekeeping across the regions, we observed a bimodal pattern of colony development and beekeeping calendar in the Oromia region of central and southern Ethiopia compared to a single peak of the development cycle and beekeeping activities in the Tigray region of northern Ethiopia (Figure 4, Table S2). In the semi-arid areas of northern Ethiopia, colony development, honey production and swarming occur from August to November, while the dearth period characterised by high rates of colony absconding extends from December to July despite the availability of a few trees that bloom during the dry period. These are consistent with previous studies reported from Oromia [61,62] and Tigray [63]. It is well-known that honey bees mainly depend on the nectar and pollen produced by flowering plants that are shaped by the local climate [24]. Therefore, the floral calendar and nectar flow of an area govern the development of honey bees and the beekeeping activities. Thus, honey harvesting is possible multiple times in a year in the evergreen areas of southwest Ethiopia [61,62], while it is mainly limited to September to November in the arid and semi-arid areas of northern Ethiopia [63].

In conclusion, colony loss rates in this survey showed a significant variation between the two Ethiopian regions, where beekeepers in Tigray sustained a markedly higher loss rate, which we attribute as being due to the war and other factors, compared to the loss rate in Oromia region and also compared to the global figures reviewed. In addition, the colony loss rate was significantly affected by beekeeping practices and honey bee health management. Moreover, the number of colony losses recorded as resulting from queen problems, natural disaster, dead colonies or empty hives is positively correlated with the number of colonies managed. Furthermore, annual colony development and management calendars in the two regions showed different patterns, where the beekeeping seasons were bimodal with short dearth periods and monomodal with a long dearth period in Oromia and Tigray regions, respectively.

## Figures and Tables

**Table 1:** Sample regions, local areas (*Kebelles*), and numbers of apiaries, beekeepers and colonies surveyed in Ethiopia using the COLOSS questionnaire tool, version 2023.

Description	Regional State		Total
	Oromia	Tigray	
No. of Kebelles	10	4	14
No. of Beekeepers	31	33	64
No. of Apiaries	35	33	68
No. of Honey bee colonies	1305	408	1713
Average no. of Colonies per apiary	42.10	12.36	26.77

**Table 2:** Beekeeping and colony management in Oromia and Tigray regions, including tests of a difference between regions.

Variables	Oromia	Tigray	Test of Difference	
			Method	*p*-Value
Colonies per beekeeper in spring 2022				
Mean	27.00	9.03	Mann–Whitney test	*p* < 0.001
Median	17.00	6.00		
Range	7–125	2–47		
Colonies per beekeeper before winter 2023				
Mean	42.10	12.36	Mann–Whitney test	*p* < 0.001
Median	27.00	8.00		
Range	10–200	2–60		
Colonies per beekeeper in spring 2023				
Mean	30.58	5.24	Mann–Whitney test	*p* < 0.001
Median	16.00	3.00		
Range	5–160	0–37		
Monitoring *Varroa destructor* (frequency)				
Yes	5	21	Fisher’s test	*p* < 0.0001
No	26	10		
Not applicable	0	2		
Treating against *V. destructor* (frequency)				
Yes	5	0	Fisher’s test	*p* < 0.0001
No	3	32		
Not applicable	23	1		
Bees with deformed wings (frequency)				
None	0	7	Fisher’s test	*p* < 0.0001
Some	31	15		
Many	0	10		
Don’t know	0	1		
Use of natural comb (frequency)				
Yes	31	14	Fisher’s test	*p* < 0.0001
No	0	19		
Purchase of external wax (frequency)				
Yes	18	30	Fisher’s test	*p* < 0.01
No	13	3		
Presence of *Vespa velutina* (frequency)				
Yes	8	29	Fisher’s test	*p* < 0.0001
No	13	4		
Don’t know	10	0		
Colony merging (frequency)				
Yes	21	6	Fisher’s test	*p* < 0.001
No	10	27		
Colony splitting (frequency)				
Yes	26	5	Fisher’s test	*p* < 0.0001
No	5	28		
Feed supplementation (frequency)				
Yes	31	17	Fisher’s test	*p* < 0.0001
No	0	16		
Queen replacement (frequency)				
Yes	19	0	Fisher’s test	*p* < 0.0001
No	12	33		
Proportional Colony loss rate (%)	24.1	66.4	Chi-squared test	*p* < 0.0001

**Table 3:** Spearman correlations between numbers of colonies managed at different times, loss rates and loss components in Ethiopia, showing the degree of association between each pair of variables. Significant associations (*p* < 0.05) are marked with an asterisk (*).

	#Colonies Before Winter	#Colonies Lost Due to Queen Problems	#Colonies Lost Due to Natural Disaster	#Empty Hives or Dead Colonies	#Colonies in Spring 2022
#Colonies lost due to queen problems	0.14				
#Colonies lost due to natural disaster	0.28 *	0.41 *			
#Empty hives or dead colonies	0.68 *	0.13	0.06		
#Colonies in spring 2022	0.92 *	0.04	0.28 *	0.52 *	
#Colonies in spring 2023	0.93 *	0.01	0.11	0.58 *	0.92 *

Note: The symbol # in the table represents the words “number of”.

## Data Availability

Additional data can be requested from the corresponding author.

## Supplementary Materials

The following supporting information can be downloaded at: https://www.mdpi.com/article/10.3390/insects15060376/s1: Table S1: Summary of best predicting quasibinomial generalised linear models for the risk of colony loss in Oromia and Tigray regions of Ethiopia, northeast Africa; Table S2: Annual honey bee colony cycle and beekeeping calendar in Oromia and Tigray regions of Ethiopia, northeast Africa.
