# Supplementary material for: High Rates of Honey Bee Colony Losses and Regional Variability in Ethiopia Based on the Standardised COLOSS 2023 Survey
Source: Insects. 2024 May 22;15(6):376. doi: 10.3390/insects15060376 (PMC11203459; doi:10.3390/insects15060376)
Supplement: Supplementary file 1 [file insects-15-00376-s001.zip › insects-2982834-supplementary.pdf]

Table S1. Summary of best predicting quasibinomial generalised linear models for the risk of colony loss in Oromia and Tigray regions of Ethiopia, northeast Africa.

Model	Predictor	Estimate of coefficient	Std. error of the estimate	t-statistic of coefficient	p-value	Overall F-statistic for variable significance	
						DF	p-value
Model for Oromia and Tigray combined	Intercept Baseline: Region Oromia, Varroa Treatment No	-1.5218	0.3155	-4.823	1.00e-05 ***	-	-
	Region Tigray	2.1922	0.3554	6.168	6.44e-08 ***	38.3700 (1,60)	5.805e-08 ***
	Varroa Treatment Not applicable	0.6849	0.3459	1.980	0.0523 .	3.2462 (2,60)	0.04585 *
	Varroa Treatment Yes	0.1198	0.3602	0.333	0.7405		
	Model fit	Null deviance: 460.89 on 63 degrees of freedom<br>Residual deviance: 200.53 on 60 degrees of freedom					
Model for Oromia	Intercept Baseline: Presence of *V. velutina* Don't know	-0.4890	0.1895	-2.580	0.01542 *	-	-
	Presence of *V. velutina* No	-0.9580	0.3007	-3.186	0.00353 **	6.4129 (2,28)	0.005095 **
	Presence of *V. velutina* Yes	-0.8697	0.2389	-3.641	0.00109 **		
	Model fit	Null deviance: 119.814 on 30 degrees of freedom<br>Residual deviance: 82.173 on 28 degrees of freedom					

Significance coding for p-values: *** $p \leq 0.001$; ** $0.001 < p \leq 0.01$; * $0.01 < p \leq 0.05$; . $0.05 < p \leq 0.1$

For Tigray region, none of the individual factors were significant in the fitted models at significance level 0.05.

Table S2. Annual honey bee colony cycle and beekeeping calendar in Oromia and Tigray regions of Ethiopia, northeast Africa.

Region	Beekeeping activities/events	Frequency of samples reporting annual beekeeping calendar on monthly basis											
		Apr	May	June	July	Aug	Sep	Oct	Nov	Dec	Jan	Feb	Mar
Tigray	Adding super	1	0	7	0	13	28	5	0	0	0	0	0
	Queen replacement	0	0	0	0	0	0	0	0	0	0	0	0
	Colony splitting	0	0	3	0	3	1	2	0	0	0	0	0
	Colony merging	0	0	1	0	0	0	1	2	0	0	2	3
	Honey harvest	0	1	1	0	0	1	14	33	8	0	2	1
	Swarming	0	0	2	0	6	21	27	9	0	0	1	1
	Absconding	10	2	0	13	21	8	0	0	8	15	18	9
	Supplementary feeding	9	5	2	3	1	0	0	0	0	4	8	9
Oromia	Adding super	13	16	1	0	0	2	12	16	11	0	0	6
	Queen replacement	4	8	5	0	0	4	8	1	0	0	0	0
	Colony splitting	7	11	8	0	0	11	17	7	0	0	0	0
	Colony merging	0	0	0	4	7	0	0	0	0	7	7	0
	Honey harvest	6	20	25	2	0	0	19	29	24	2	6	6
	Swarming	7	31	23	0	0	14	28	19	0	0	0	0
	Absconding	14	13	3	0	0	0	0	0	1	5	14	13
	Supplementary feeding	25	1	0	1	28	30	6	0	0	28	31	28
